# Pulmonary arteriovenous malformations as an unusual initial manifestation of gestational trophoblastic neoplasia: a case report from Saudi Arabia

**DOI:** 10.1093/jscr/rjag333

**Published:** 2026-04-28

**Authors:** Modhi Mohammed AlJumah, Hani Abdulmohsen AlHalal, Omar Khalid alzaydan

**Affiliations:** Obstetric and Gynecology Resident, King Khalid University Hospital, King Saud University Medical City, Riyadh 12372, Saudi Arabia; Gynecologic Oncologist Consultant, King Khalid University Hospital, King Saud University Medical City, Riyadh 12372, Saudi Arabia; Obstetric and Gynecology Consultant, King Khalid University Hospital, King Saud University Medical City, Riyadh 12372, Saudi Arabia

**Keywords:** gestational trophoblastic tumor and arteriovenous malformations

## Abstract

Pulmonary arteriovenous malformations (PAVMs) are abnormal communications between an artery and a vein without an intervening capillary component. Most PAVMs are congenital, caused by hereditary hemorrhagic telangiectasia, but around 20% are acquired. The most common presenting symptom is dyspnoea on exertion, but patients can be completely asymptomatic. Furthermore, Gestational trophoblastic neoplasia (GTN) is a group of rare tumors that arise from abnormal growth of trophoblastic tissue which includes invasive mole, choriocarcinoma, placental site trophoblastic tumor, and epithelioid trophoblastic tumor can progress, invade, metastasize, and lead to death if left untreated. In the majority of GTNs, the disease is limited to the uterus where the abnormal trophoblast proliferation and localized hCG production may lead to focal vascular changes, including the formation of AVMs. Most patients with GTN present with abnormal vaginal bleeding but they can also present with bleeding from metastatic sites. Symptoms of pulmonary metastasis from GTN often include cough, hemoptysis, and chest pain. However, PAVMs are not typically listed as a direct consequence of this metastatic spread. Here, we report a rare case of metastatic GTN initially presented with pulmonary vascular malformation.

## Background

Pulmonary arteriovenous malformations (PAVMs) are abnormal communications between an artery and a vein without an intervening capillary component [1]. Most of PAVMs are congenital caused by hereditary hemorrhagic telangiectasia (HHT). However, around 20% are acquired [2–4]. The most common presenting symptom is dyspnoea on exertion which is seen in 67% of patients [5–8] hemoptysis and haemothorax may also occur [9]. Gestational trophoblastic neoplasia (GTN) can progress, invade, metastasize, and lead to death if left untreated [10, 11]. These tumors are extremely sensitive to chemotherapy and high cure rates approaching 100% can be expected. The disease is usually limited to the uterus, where the human chorionic production can lead to vascular changes including the formation of arteriovenous malformations [12]. Here, we report a case of metastatic GTN initially presented with PAVM. This case report raises questions for further discourse and research on PAVM and its association with GTN.

## Case presentation

A 21 year-old lady was referred from another region in Saudi Arabia as a case of hereditary hemorrhagic telangiectasia (Osler–Weber–Rendu disease) CT done in the referring hospital which showed: multiple pulmonary arteriovenous malformation with partial thrombosis in both lungs as well as enlarged liver size with multiple hypodense lesions suggestive of hemangioma and moderate free fluid in the pelvis. She is Para 2, all were spontaneous vaginal delivery, last delivery was five months prior to her referral. Obstetric and gynecological team was consulted by General Medicine as her Quantitative BHCG level was done routinely prior imaging turned out to be 25 774. The patient upon her presentation was having generalized abdominal pain, palpitation and she was having headache along with blurry vision.

**Figure 1:**
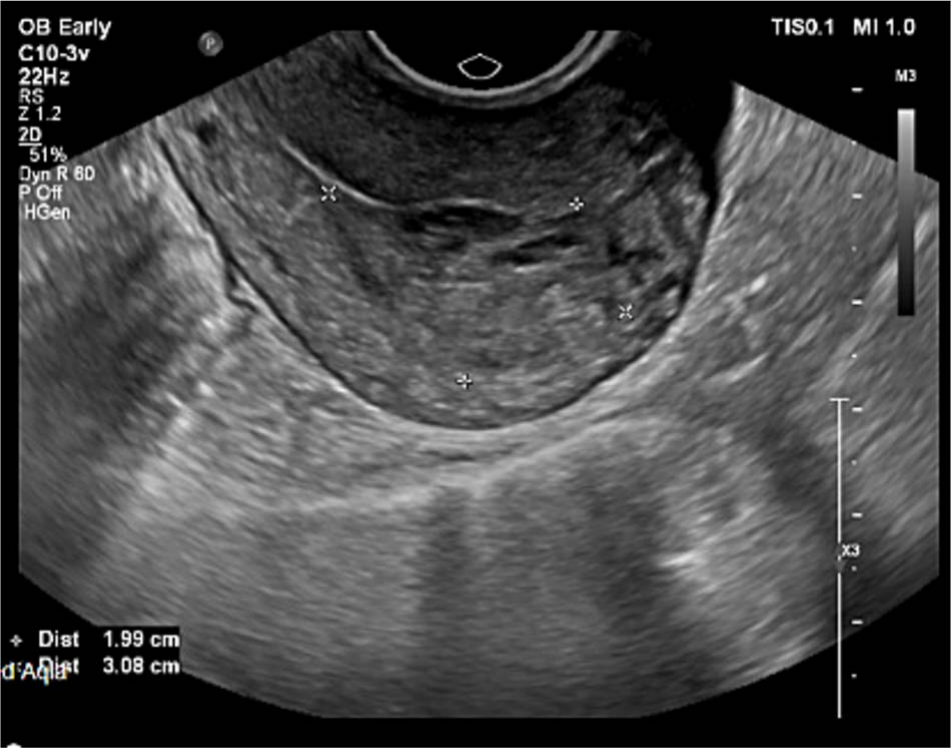
Pelvic ultrasonography.

**Figure 2:**
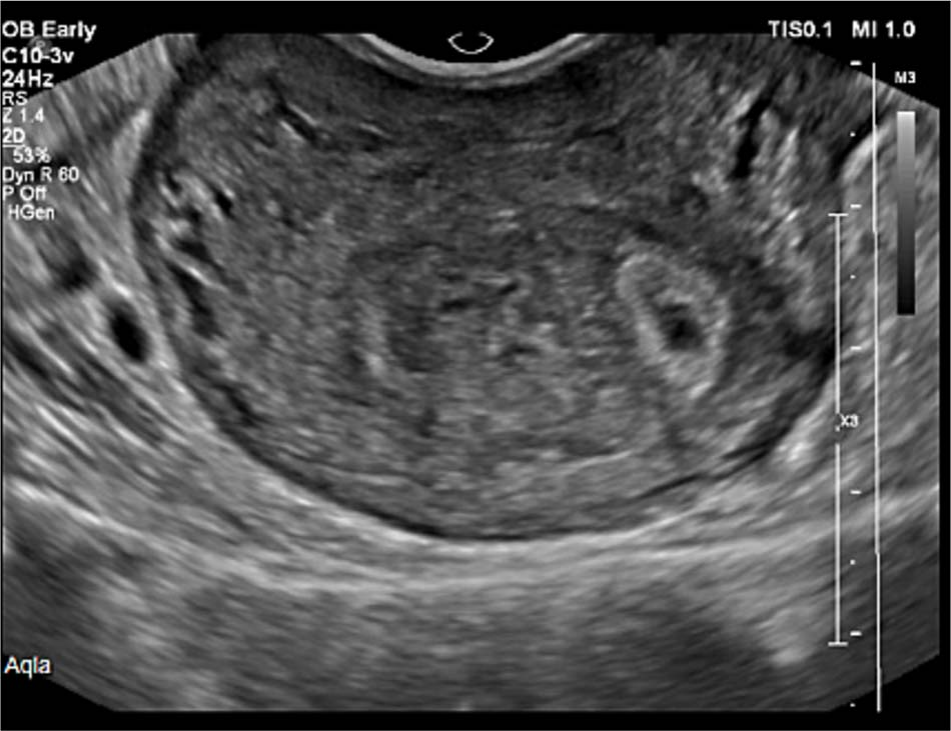
Pelvic ultrasonography.

All malignancy work-up and Tumor markers turned out to be unremarkable, quantitative BHCG has repeated which was increased to 48 790. Gynecological USG has done which showed in Figs 1 and  2 a cystic mass measured 3 × 2 cm with minimal vascularity flow in Doppler was strongly associated with GTD.

**Figure 3:**
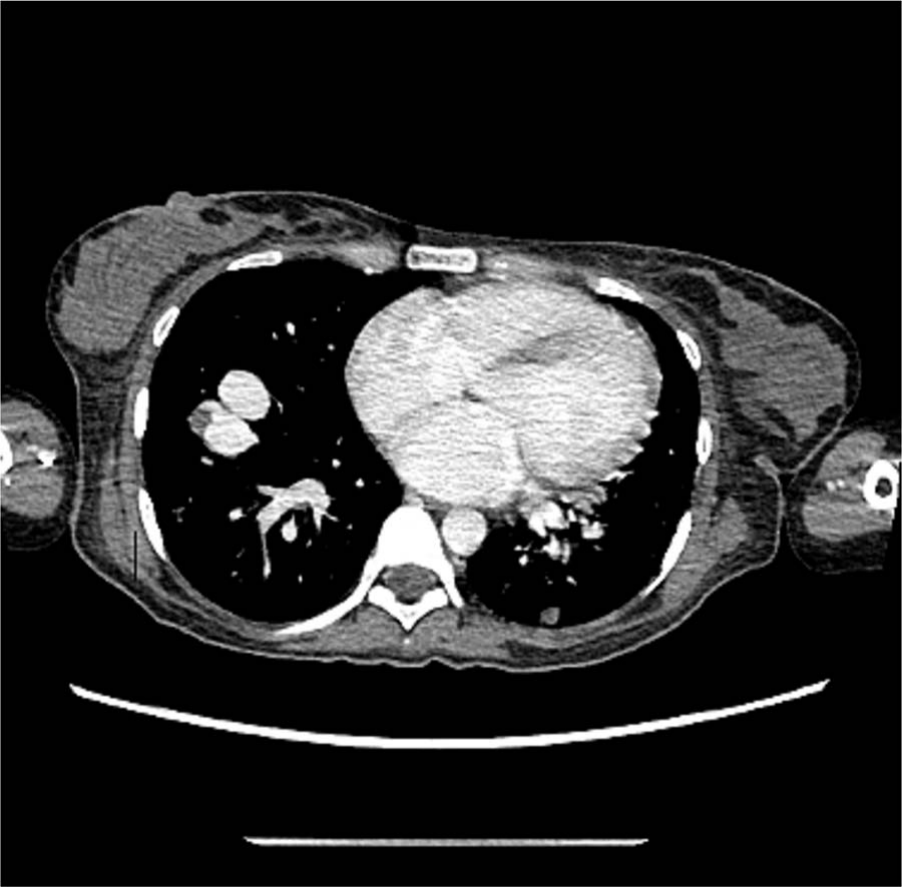
CT of the chest showed multiple AVMs.

**Figure 4:**
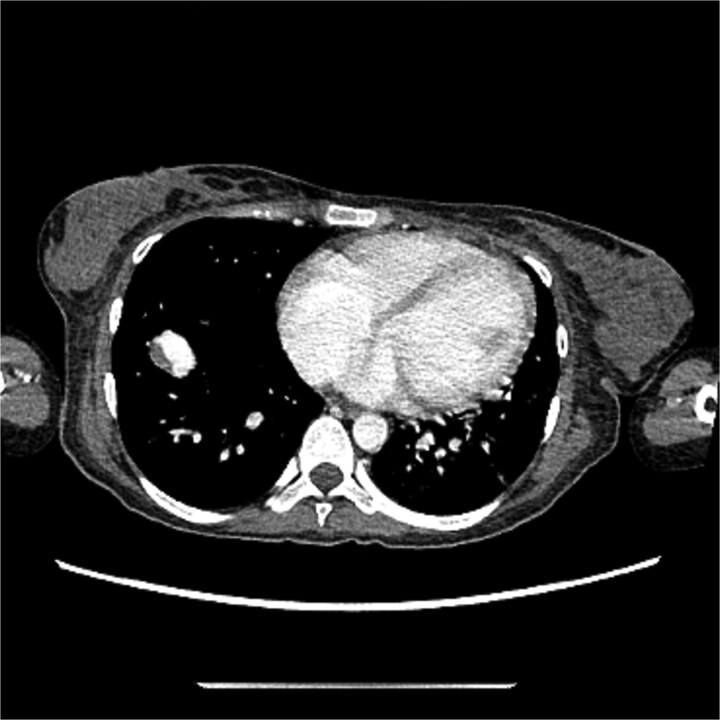
CT of the chest showed multiple AVMs.

Figures 3 and 4 illustrate the computerized tomography (CT) scan of the chest which showed multiple bilateral lung hyper vascular lesions with feeding vessels seen arising from the pulmonary artery with a venous drainage, largest seen in the right lower lobe measuring 2.7 × 2.2 cm some of which demonstrate partial thrombosis, these lesions are suggestive of pulmonary arteriovenous malformations.

**Figure 5:**
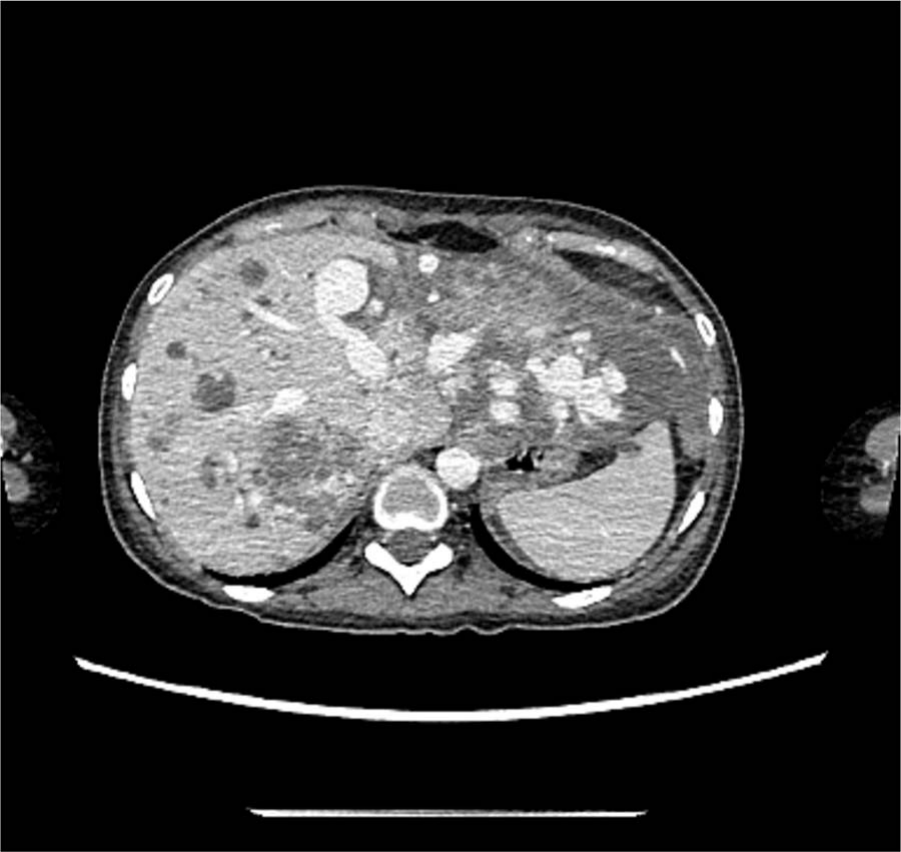
CT of the liver showed several AVMs and thrombosis.

**Figure 6:**
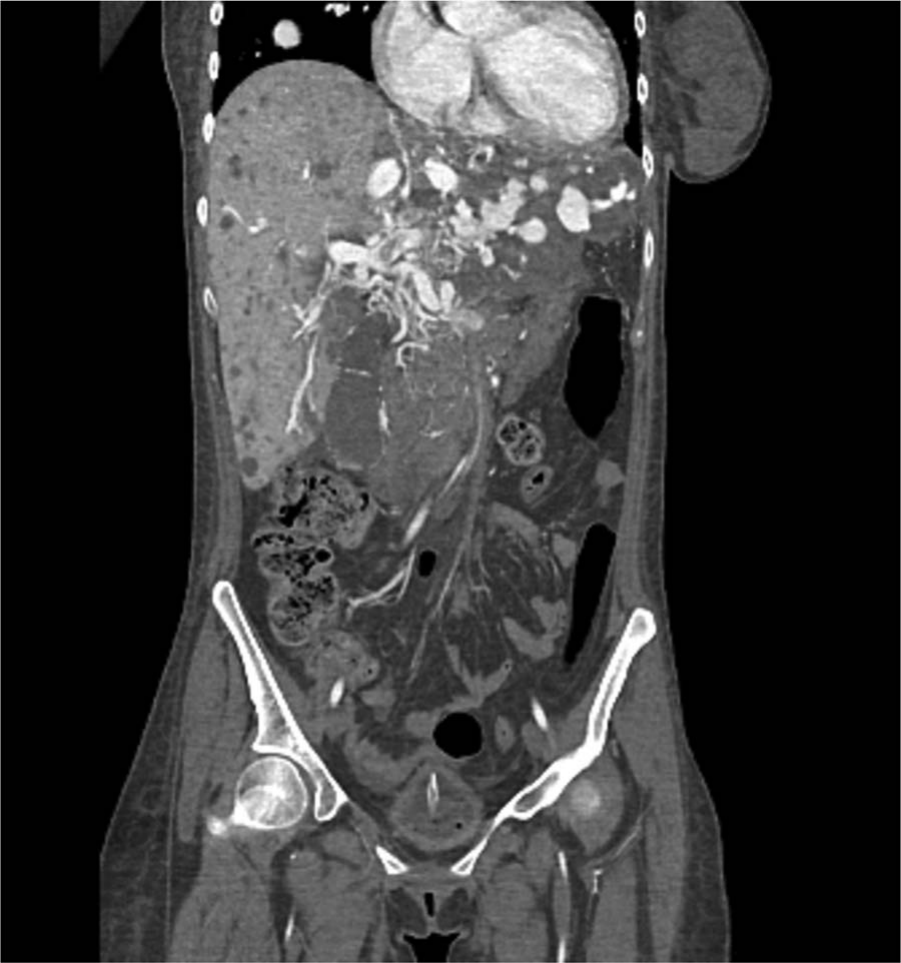
CT of the liver showed several AVMs and thrombosis.

The liver shows multiple hepatic and portal vein arteriovenous malformations mainly in the left lobe. Some of the AV malformations showed filing defects in keeping with partial thrombosis. The proximal portal and hepatic veins are patent. The hepatic artery is patent as well which demonstrated in Figs 5 and 6.

### CT brain reveals no distant metastasis

**Figure 7:**
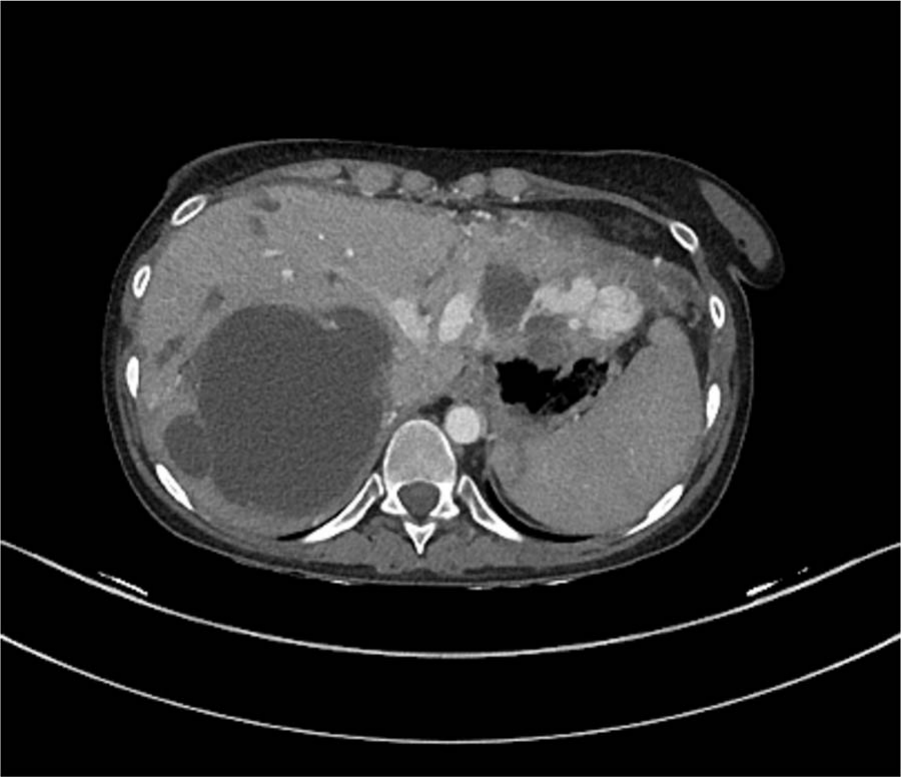
Multiple contrast extravasations seen which represent multiple active bleeding.

Day 5 in her hospital stay we noticed dropping in her hemoglobin level from 10 to 8, an urgent CT was made to see if there is any bleeding from the AVMs. However, shown in Fig. 7, multiple contrast extravasations seen at right hepatic lobe and blood pooling seen which represent multiple active bleeding compressing the inferior vena cava and right adrenal gland along with portal vein has compressed by the hematoma, patient underwent IR embolization of right hepatic artery.

The diagnosis was made to be GTN with widely metastasis (Stage-IV) represented as arteriovenous malformations with high score (prognostic score > 12). As per the tumor board the finding in the lung and the liver represents metastasis in the form of AVM. The patient was started on low dose induction chemotherapy with intravenous etoposide, cisplatin for 2 courses prior to starting EMA/CO protocol.

**Figure 8:**
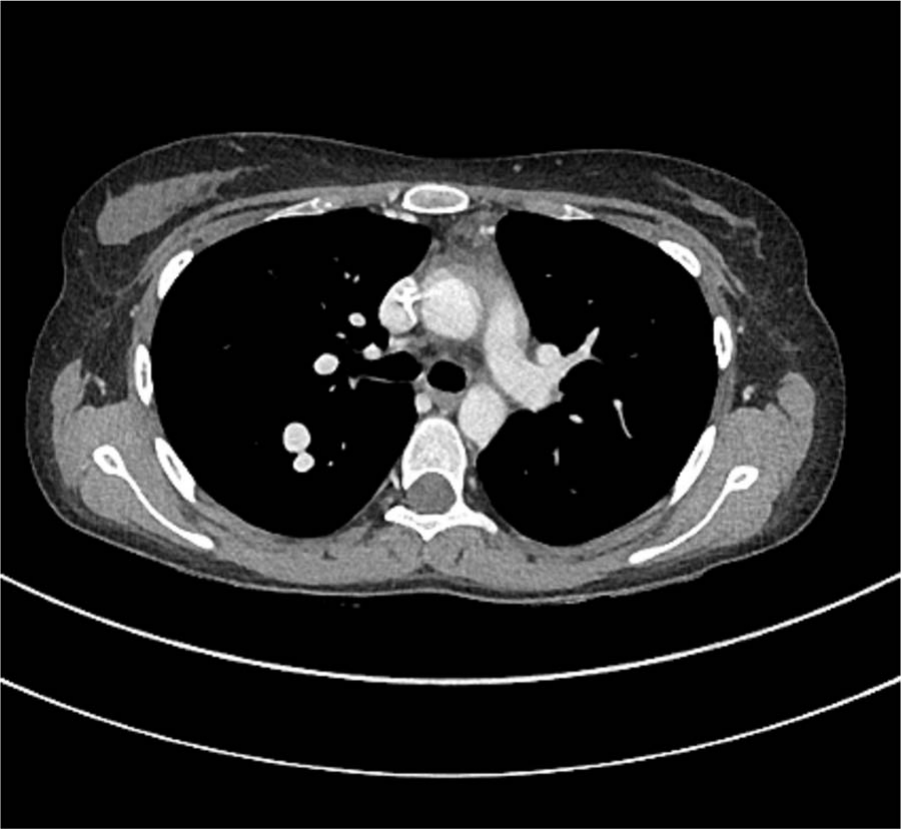
CT of the chest post-receiving chemotherapy by three months.

**Figure 9:**
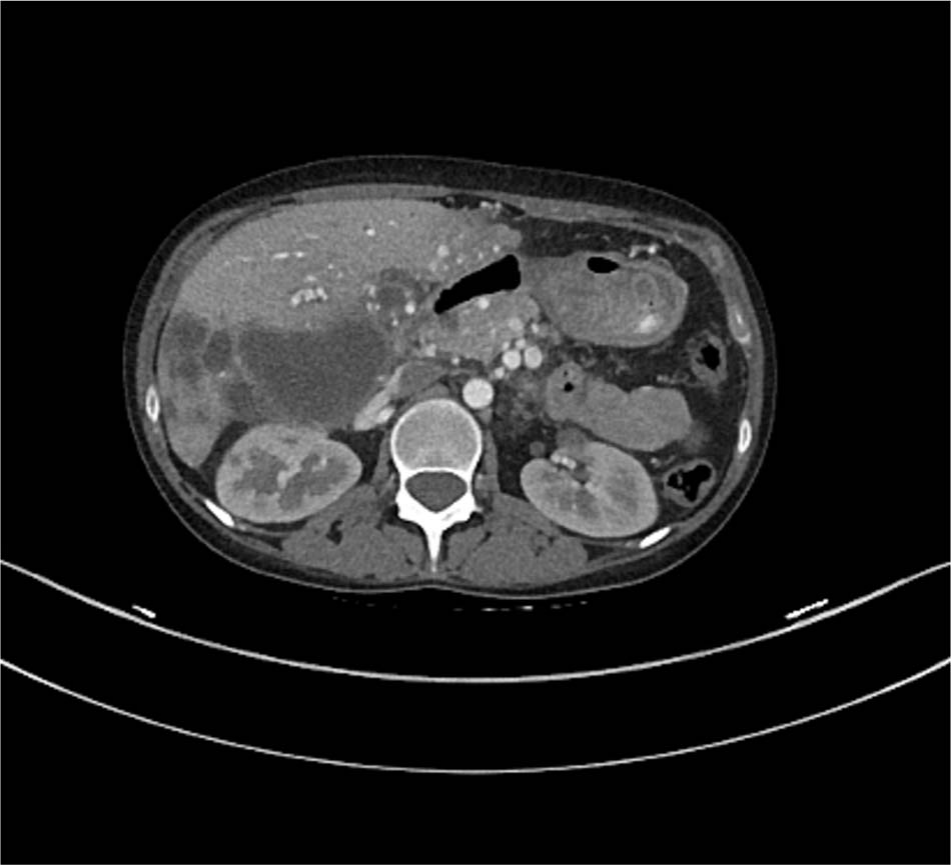
CT of the liver post-receiving chemotherapy by three months.

The images have repeated after almost 3 months after receiving chemotherapy as shown in Figs 8 and 9. Shows regression of most AV malformations in both lungs in particular on the left also improvement of thrombotic component in particular the AVM in anterior segment right lower lobe.

## Discussion

GTN is a rare but highly curable form. Majority of cases occur as a result of a complete molar pregnancy and are characterized as hCG producing, highly vascular and chemo-sensitive tumors. The effect of the tumour-derived hCG on angiogenesis is well documented [13] and AVMs in the uterus are a relatively frequent event. Furthermore, GTN has the potential to metastases to other anatomical sites that are highly vascular and AVMs formation that can lead to life-threatening hemorrhage. Although the formation of Pulmonary; AVMs secondary to metastatic GTN is extremely rare, with only few reported cases and mostly are a cases of choriocarcinoma. Once pulmonary AVMs are diagnosed it is suggested that urgent referral for embolization should be considered to reduce the risk of bleeding [14]. In this case the CT abdomen showed multiple hepatic hypo and heterogeneously hyper-dense masses and hepatic AVMs with multiple contrast extravasations. Patient underwent urgent IR US guided liver embolization; the right hepatic artery was catheterized and embolized. Chemotherapy is an effective treatment for GTN and has allowed complete remission even when there is evidence of metastatic disease [10]. A Patients with high-risk (FIGO ≥7) frequently receive etoposide, methotrexate, and dactinomycin alternating weekly with cyclophosphamide and vincristine (EMA/CO) [15]. In this case the patient was started on low dose induction chemotherapy with intravenous etoposide 100 mg/m2/day and intravenous cisplatin 20 mg/m2/day on Day 1 and 2 every 7 days for 1–2 courses prior to starting EMA/CO protocol. Finally, This case report will add a value by highlighting the regional differences in presentation, diagnosis and management of GTN and it will raise the awareness to help in early detection.

## Conclusion

Pulmonary AVMs are rare but potentially life threatening complications secondary to GTN. In patients with abnormal HCG levels and a pulmonary AVM without meeting any other clinical criteria for HHT there should be heightened concern for GTN as the diagnosis. Awareness of this association is crucial for early detection and appropriate treatment.
